# Endoscopic Thyroidectomy for Large-Sized Goiters: Merits of the Axillo-Breast Approach with Gas Insufflation

**DOI:** 10.1155/2024/9487076

**Published:** 2024-02-06

**Authors:** Islam A. Elzahaby, Essam Attia Ali, Ahmed Mohammed Farid, Mohamed Abd El Ghaffar Saleh, Ahmed Abdallah

**Affiliations:** Mansoura University, Mansoura, Egypt

## Abstract

**Background:**

Several minimal access approaches to the thyroid gland have been widely applied; nevertheless, such approaches are still challenging when dealing with large-sized thyroid nodules or goiters. We hereby evaluated the outcomes and highlighted the merits of endoscopic axillo-breast hemithyroidectomy (EABH) for large-sized unilateral goiters.

**Methods:**

Patients underwent EABH for unilateral large thyroid nodules ≥6 cm in its greatest dimension or unilateral large goiter (≥60 ml sonographic volume) whatever the size of its contained nodules were identified from a prospectively maintained database. Their demographic data, clinicopathological profiles, and surgical and esthetic outcomes are reported and analyzed.

**Results:**

Over a 2-year period, 33 patients matched the selection criteria. Their mean age was 34.75 ± 11.39 years. There were 30 women and 3 men. The majority of nodules were radiologically TIRADS3 and cytologically Bethesda 3. The mean sonographic dominant nodule greatest dimension was 5.29 ± 1.48 cm (range: 3–9.5 cm). The mean sonographic volume of the pathological lobe was 101.86 ± 54.45 ml (range: 60.11–236.88 ml). All cases were completed endoscopically with no conversion to open. The mean operative time was 110.76 ± 18.75 minutes. No significant postoperative complications were reported except for one case with temporary vocal cord paresis. Most (87.9%) of the patients were extremely satisfied with the procedure.

**Conclusion:**

EABH with our suggested key steps could be considered an effective valid approach for unilateral large goiters in trained hands and in patients desirous for cosmesis.

## 1. Introduction

Minimal access thyroidectomy approaches have been widely practiced all over the world, with the extracervical approaches being more applied and desirable, owing to its higher esthetic outcome (small, hidden scars outside the neck region) and precision (good illumination and magnified view) compared to conventional open approaches [1–3].

One notable limitation of all endoscopic thyroidectomy approaches is dealing with large goiters because it is intuitively difficult to safely and efficiently handle these large lesions in the narrow endoscopic working space [4, 5].

Several studies reported the safety and feasibility of different endoscopic and robotic approaches in removing large-sized goiters and/or thyroid nodules larger than 4–6 cm in its greatest dimensions [4, 6–8].

In this study, we aimed firstly at analyzing the surgical and esthetic outcomes of endoscopic axillo-breast hemithyroidectomy (EABH) with gas insufflation for patients with unilateral large thyroid nodules ≥6 cm in its greatest dimension or with unilateral large goiters (≥60 ml sonographic volume) whatever the size of its contained nodules and secondly to highlight the merits of the axillo-breast approach in handling these large-sized goiters and nodules.

## 2. Patients and Methods

This is a retrospective study of a prospectively maintained database in two tertiary Egyptian cancer centers (Mansoura University Oncology Center and Meet Ghamr oncology Center) between 2021 and 2023. The selection criteria were patients younger than 65 years and who do not have significant medical comorbidities, patients without previous neck surgery or irradiation, patients opting for scareless neck thyroidectomy, and euthyroid patients with unilateral thyroid nodules ≥6 cm in its greatest dimension or unilateral large goiters (≥60 ml sonographic volume) whatever the size of its contained nodules. In all selected patients, a thorough preoperative evaluation by history, physical examination, thyroid function tests, high resolution neck ultrasound (US) for the accurate diameters and criteria of the nodules and lobe volume, and ultrasound guided biopsy (fine needle aspiration cytology) was accomplished. All clinical procedures were conducted in accordance with the guidelines of our ethics committee and after obtaining written consent from all the patients after a detailed discussion of the planned procedure and its potential sequelae (IRP approval code: R.23.03.2132)

### 2.1. Surgical Technique

**Figure 1:**
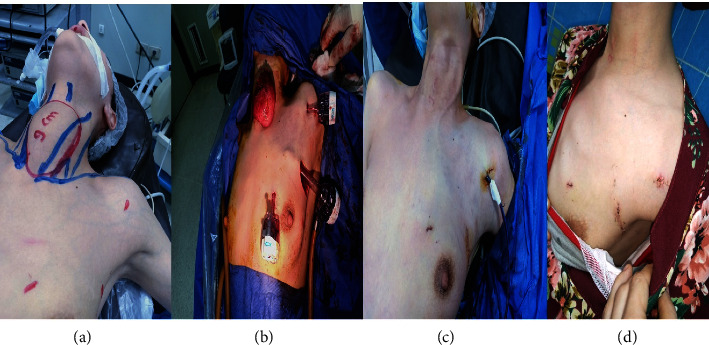
EABH for female patient with huge left thyroid lobe. (a) Preoperative view, (b) view after lobe retrieval, (c) immediate postoperative view, and (d) two weeks postoperative view.

**Figure 2:**
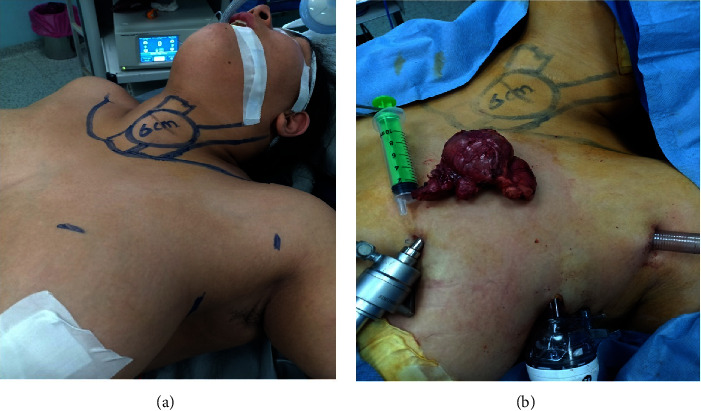
EABH for a female patient with large left lobe multinodular goiter: (a) preoperative markings and (b) view showing the large left lobe after retrieval.

**Figure 3:**
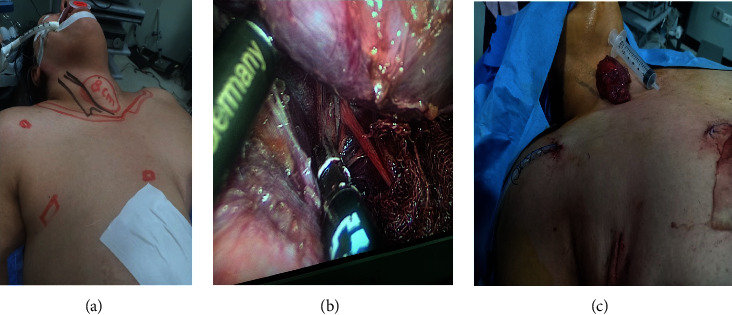
EABH for female patient with large right lobe multinodular goiter. (a) Preoperative view, (b) intraoperative view of right recurrent laryngeal nerve, and (c) view after right lobe retrieval.

**Figure 4:**
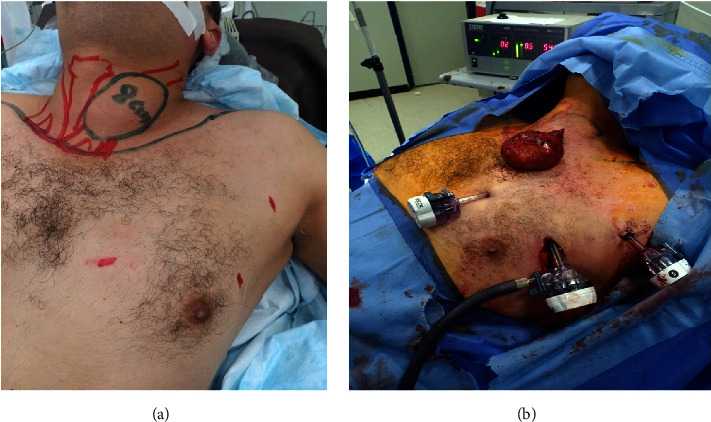
EABH for male patient with large left lobe goiter. (a) Preoperative view and (b) view after left lobe retrieval.

**Figure 5:**
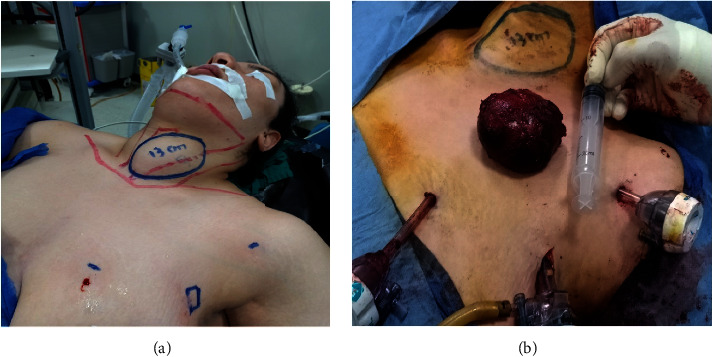
EABH for large left lobe multinodular goiter in a female patient: (a) view of the preoperative markings and (b) view of of the ports and the large left lobe after retrieval.

**Figure 6:**
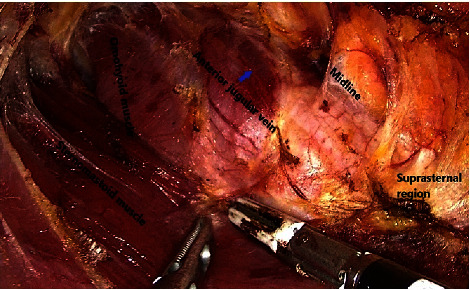
Wide working space creation for large goiters.

**Figure 7:**
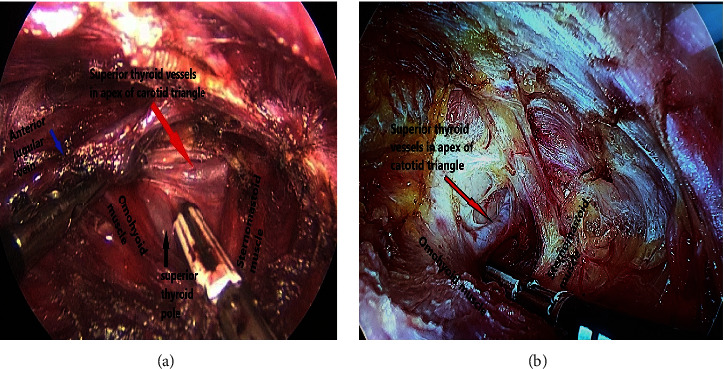
Premature dissection of the superior thyroid vessels in the apex of carotid triangles in two different patients of the study.

**Figure 8:**
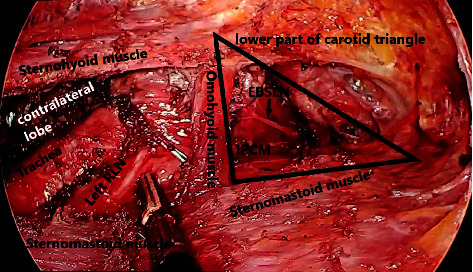
Intraoperative view showing left recurrent laryngeal nerve (RLN) and external branch of superior laryngeal nerve (EBSLN), inferior pharyngeal constrictor muscle (IPCM), and the carotid triangle.

All selected patients had EABH performed following the same steps described by the senior author in his previous publications [3, 9] **(**Figures 1–5), with the following additional key steps:

1. Wide adequate working space should be created and extended for 2-3 cm beyond the visible borders of the goiter to give room for goiter grasping and manipulation (Figure 6).
2. The key step is premature sealing and dividing of the superior vascular pedicle in the apex of the carotid triangle before attempting lobe dissection. This key step is performed early in the procedure, after the creation of the adequate working space, in which the apex of the carotid triangle between the lateral border of the ipsilateral omohyoid muscle and the medial border of the ipsilateral sternomastoid muscle is dissected till identification of the superior thyroid vessels running towards the upper thyroid pole where these vessels are carefully sealed and divided facilitating further lobe delivery and mobilization without the risk of avulsion or tear of the superior thyroid vascular pedicle and without the need to divide the strap muscles horizontally (Figure 7). After finishing this step, the lobe is then accessed through the space between the sternomastoid and sternohyoid muscles and then in the deeper plane between the omohyoid and sternohyoid muscles as described before in our previous publication [10] (Figure 8).
3. 10 mm camera port is widened in all cases to allow for specimen retrieval.
4. A suction drain was inserted in the operative bed and left in place for 4 days, and the total amount of drainage is calculated.

The patients' baseline data, clinicopathological profiles, surgical outcomes including operative time, estimated blood loss, hospital stay, and perioperative complications (including conversion to open, recurrent laryngeal nerve affection, paresthesia, postoperative pain using a subjective pain score analysis from 1 to 10 where 1 is no pain and 10 worst pain ever, wound infection, hematoma, seroma formation, neck contracture, hypercapnia, surgical emphysema, and gas embolism), esthetic outcomes (two months postoperatively), and the recurrence rates during one year follow-up period were gathered and analyzed.

The esthetic outcome was evaluated two months postoperatively in the outpatient clinic by reporting the degree of patient satisfaction using a simple scoring system (1 = extremely satisfied, 2 = satisfied, 3 = acceptable, and 4 = dissatisfied).

Patient data were analyzed using SPSS version 22 (SPSS Inc., Chicago, IL, USA). Continuous variables were presented as means when symmetrical or as medians and ranges when asymmetrical. Categorical variables were presented as proportions. A *p* value <0.05 was considered significant.

## 3. Results

Thirty-three patients were included, and the patients' baseline criteria, clinicopathological characteristics, and surgical and esthetic outcomes are presented in Table 1. Mean age was 34.75 ± 11.39 years (range: 20–61 years). There were 30 women and 3 men (F : M = 10 : 1). Two patients were smokers, four were hypertensive, three were having controlled diabetes mellitus, and three women were morbidly obese. In all patients, there was clinical unilateral thyroid enlargement (right sided in 19 patients and left sided in 14 patients). The mean duration of symptoms was 30.3 ± 20.5 months. Most of the patients (29 patients, 87.87%) were euthyroid, 3 patients were having subclinical hypothyroidism, and one patient was having controlled toxic adenoma. The preoperative neck sonography revealed solitary thyroid nodules in 27 patients (81.8% of the patients) and unilateral multiple nodules in 6 patients (18.2%) without any significant cervical lymph node enlargement. In most of the patients (22 patients, 66.7%), the thyroid nodules were categorized as TIRADS 3. The mean sonographic dominant nodule greatest dimension was 5.29 ± 1.48 cm (range: 3–9.5 cm), and the mean sonographic volume of the pathological lobe was 101.86 ± 54.45 ml (range: 54.67–236.88 ml). In twenty-nine patients, the sonographic volume of the pathological lobe exceeded 60 ml; nevertheless, only ten patients had dominant nodule ≥6 cm in its greatest dimension. Preoperative fine needle aspiration cytology revealed Bethesda category 2 in twenty-two patients (66.8%), category 3 in seven patients (21.2%), and category 4 in three patients (9%). In one patient (3%), the FNAC was hemorrhagic (Bethesda category 1) twice, and for this particular patient, diagnostic lobectomy/hemithyroidectomy was decided rather than obtaining a third FNAC.

EABH was successfully performed in all cases (right hemithyroidectomy in 19 patients and left hemithyroidectomy in 14 patients) with no conversion to an open approach. All procedures were performed by the same surgeon (the senior author). Premature sealing and dividing the superior thyroid pedicle at the apex of the carotid triangle was attempted in all cases. The 10 mm camera port was widened in all cases to allow specimen retrieval. The mean operative time was 110.76 ± 18.75 minutes (range: 95–165 minutes), and the mean operative blood loss was 16.97 ± 7.28 ml (range: 10–35 ml). All patients were discharged in the second postoperative day. The drains were removed on the fourth postoperative day, and the mean amount of drainage was 86.13 ± 18.00 ml (range: 50–110 ml). Recurrent laryngeal nerve (RLN) was identified and preserved in all cases; however, the external branch of the superior laryngeal nerve (EBSLN) could be identified in ten cases (30.30%) only. The postoperative course was uneventful, and all patients were discharged in the second postoperative day without the need for further hospitalization. There were no CO_2_ insufflation-related complications such as hypercapnia, acidosis, surgical emphysema, and gas embolism. There were no reported significant postoperative complications apart from a single case with temporary RLN affection (in the form of temporary hoarseness of voice and choking) which was resolved spontaneously within 3 weeks. Cellulitis at the camera port was observed in three cases (9%), two cases (6%) developed seroma in the ipsilateral upper chest wall which was effectively managed by aspiration, and five patients (15.15%) suffered from temporary ipsilateral neck contracture which was resolved gradually within 1-2 months postoperatively. None (0%) of the patients had postoperative hemorrhage or hematoma.

The mean postoperative subjective pain scores were highest in the first postoperative day (4.03) and then decreased gradually until the seventh postoperative day (0.35) after which no pain was experienced in any of the patients of the study. Temporary paresthesia in the ipsilateral upper anterior chest wall and neck was subjectively reported in 25 patients (75.8%), which was resolved gradually within 3-4 months postoperatively.

Completion thyroidectomy without lymphadenectomy was required for two cases (6%) in whom postoperative pathology revealed multifocal papillary microcarcinoma and follicular carcinoma (4 cm in diameter and with 4 vessels invasion). The completion thyroidectomy was successfully performed endoscopically via the contralateral virgin side.

Two patients (6%) developed postoperative hypertrophic scars at the camera port; nevertheless, most of the patients (87.9%) reported extreme satisfaction with the procedure.

None (0%) of our patients developed any locoregional or distant recurrence over one year follow-up period.

## 4. Discussion

Endoscopic thyroidectomy (ET) has evolved dramatically in the past few decades and became a comparable alternative to open conventional thyroidectomy [9, 11, 12]. However, the technique, approaches, and criteria for indications and contraindications are still in the process of continuous research, evolution, and refinement. The extracervical remote access approaches (such as trans-axillary, breast, axillo-breast, anterior chest wall, retroauricular, and trans-oral approaches) [3, 13–17] have emerged as a more esthetic modality compared to the direct cervical approaches as the former avoided any neck incisions. The technique itself witnessed various refinements with the description of specific key steps and even a critical view of safety [10, 17, 18]. The indications have extended to include not only benign lesions but also early thyroid malignancy [19–21]. Furthermore, most of the limitations have been overcome, such as the size limit, thyrotoxicosis, and feasibility of central and lateral lymph node dissection [8, 22, 23].

In its early phases, minimal access thyroidectomy was limited to small-sized goiters (less than 4 cm in its greatest dimension or 25 ml volume) owing to the intuitive difficulty in grasping and manipulating large-sized lesions within the small endoscopic working space and the potential risk of tumor spillage on condition that the capsule is ruptured [24–26]. Nevertheless, with the continuous evolution and refinement of the technique, several researchers have reported the safety and efficacy of endoscopic and robotic-assisted thyroidectomy for large-sized goiters ≥4 cm in diameter or 25 ml sonographic volume [24, 27].

In his two-case series, Puntambekar successfully removed thyroid nodules up to 8.5 cm in diameter and thyroid volume up to 112 ml for hemithyroidectomy and 160 ml for total thyroidectomy via an endoscopic anterior chest wall approach [7, 28]. Duncan successfully performed trans-axillary endoscopic total thyroidectomy for three cases with large multinodular goiters ranging from 87 to 105 grams, and every lobe was about 7 cm in its greatest dimension [5].

Furthermore, Johri and his colleagues emphasized the efficacy of endoscopic axillo-bilateral breast (ABB) and bilateral axillo-breast (BAB) approaches in thyroidectomy (hemi and total thyroidectomy) for 39 large goiters ranging from 6 to 11 cm in the greatest dimension (mean was 6.7 ± 1.1 cm) and from 18 to 253 grams in weight (mean was 62.3 ± 51.3 grams) [4].

In this study, we reported the outcomes of EABH for 33 patients with large-sized goiters which were completed successfully with no conversion to open and with no significant postoperative complications apart from a single case with unilateral temporary RLN palsy. The mean dominant nodule greatest dimension was 5.29 ± 1.48 cm (range: 3–9.5 cm), and the mean sonographic volume of the pathological lobe was 101.86 ± 54.45 ml (range: 60.11–236.88 ml).

To the best of our knowledge, no previous reports stated successful endoscopic hemithyroidectomy for a thyroid nodule 9.5 cm in diameter and/or a thyroid lobe of 236.88 ml sonographic volume.

Based on this study and our experience with the axillo-breast approach, we recommend this approach with the proposed additional key steps when removing large goiters as it provides a lateral route of access to the thyroid lobe which has the following merits:

1. It ensures excellent exposure and preservation of RLN, parathyroid glands, and EBSLN as well as easy and efficient control of the middle thyroid vein if found
2. It overcomes the technical difficulty in accessing the upper pole in large goiter [4] with the ability to prematurely control the superior thyroid pedicle within the apex of the carotid triangle
3. It decreases the necessity for strap muscle division to deliver the large goiter, which could lead to intraoperative troublesome bleeding [4] and impaired postoperative voice quality
4. The gap created between the muscles is not closed and acts as a safety valve to avoid life-threatening hematoma where postoperative hemorrhage occurred
5. An important advantage of this lateral route extracervical approach is the presence of the camera port in a hidden location in the axilla which can be enlarged to retrieve the large-sized goiter without significant adverse esthetic outcome unlike the anterior chest wall or transoral approaches

Moreover, we recommend that access to the large thyroid lobe should be performed through the plane between the sternomastoid and sternohyoid muscles and then in the deeper plane between the omohyoid and sternohyoid muscles, not between the two heads of the sternomastoid because the separation gap between the two heads is often small to manipulate the large goiter and to avoid the potential risk of injury to the brachial plexus.

It is worth mentioning that applying other endoscopic thyroidectomy approaches will be very challenging when dealing with large goiter. That in direct cervical endoscopic approaches, the instrument handling and maneuverability will be so difficult and field visualization will be hindered by the large goiter; moreover, the lobe retrieval will require large neck incision which abolishes the advantage of the endoscope. In the other extracervical endoscopic approaches such as transoral and chest wall, the midline route of access to the thyroid is used which makes lobe delivery and access to the upper pole much more difficult in large goiters and will require significant pretracheal muscle division; moreover, the lobe retrieval will require a large vestibular incision (which may jeopardize the mental nerve) or a large midline sternal incision (with subsequent potential keloid or hypertrophic scar).

Some limitations were considered in our study: firstly the retrospective nature of the study and secondly the small sample size, which may restrict the generalizability of our findings. To address this limitation, we have been conducting a prospective study involving a larger cohort of patients to compare our results with conventional open approach and to evaluate patient satisfaction and esthetic outcomes. Thirdly, the follow-up period of 12 months in our study may be considered relatively short for assessing long-term oncological safety. We have been extending the follow-up duration to gather more comprehensive and reliable long-term outcomes.

## 5. Conclusion

Based on our positive experience in EABH and owing to its advantages and favorable outcomes, it could be an effective valid approach for unilateral large goiters in trained hands and in patients desirous for cosmesis.

## Figures and Tables

**Table 1:** Demographic, clinicopathological, and perioperative characteristics of the patients of the study.

Parameter	Value
Age:	
Range	20–61 years
Mean + SD	34.75 ± 11.39 years
Sex:	
Female	30
Male	3
Comorbidities:	
Smoking	2
Hypertension	4
Diabetes	3
Morbid obesity	3
Other chronic illnesses such as hepatic, cardiac, and renal	0
Preoperative thyroid function:	
Euthyroid	29
Clinical hypothyroidism	3
Controlled toxic adenoma	1
Preoperative sonographic characters of nodule(s):	
Side (right/left)	19/14
Number (single/multiple)	27/6
Dominant nodule greatest dimension:	
Range	3–9.5 cm
Mean ± SD	5.29 ± 1.48 cm
TIRAD scoring (T3/T4a/T4b)	(22/8/3)
Preoperative sonographic volume of pathological lobe in ml:	
Range	60.11–236.88 ml
Mean	101.86 ± 54.45 ml
Preoperative FNAC:	
Bethesda 1 (no, %)	1 (3%)
Bethesda 2 (no, %)	22 (66.8%)
Bethesda 3 (no, %)	7 (21.2%)
Bethesda 4 (no, %)	3 (9%)
Operative time:	
Range	95–165 minutes
Mean + SD	110.76 ± 18.75 minutes
Estimated blood loss:	
Range	10–35 ml
Mean + SD	16.97 ± 7.28 ml
Total drain output:	
Range	50–110 ml
Mean + SD	86.13 ± 18.00 ml
RLN visualization (no, %)	All (33) patients
EBSLN visualization (no, %)	10 cases (30.30%)
RLN affection:	
Temporary (no)	One patient
Permanent (no)	No patient
Hospital stays:	
2 days (no)	All (33) patients
More than 2 days (no)	0 patient
Mean drain output over the first 4 PO days (ml):	
Range	50–100 ml
Mean + SD	76.36 ± 15.58 ml
Paresthesia in neck/upper anterior chest wall:	
No paresthesia (no)	8 (24.24%)
Temporary (no)	25 (75.75%)
Permanent (no)	0 (0%)
Mean postoperative subjective pain scores:	
POD1	4.03
POD2	2.66
POD3	1.33
POD7	0.35
Neck contracture:	
No neck contracture (no)	28
Temporary (no)	5
Permanent (no)	0
Seroma	2 cases, managed by aspiration
Intraoperative hemorrhage or postoperative hematoma	0
Wound complications:	
Cellulitis	3
Wound gapping	0
Infection with discharge	0
Hypertrophic scar	2
Keloid	0
Completion thyroidectomy:	
Required (no)	2
No required (no)	31
Postoperative pathology:	
Benign:	
Thyroid follicular nodular disease	25
Follicular adenoma	3
Low risk neoplasms:	
NIFTP	2
Malignant:	
Unifocal papillary microcarcinoma	2
Multifocal papillary microcarcinoma	1
Follicular carcinoma (reaching the capsule with 4 vessels invasion)	1
Patient satisfaction:	
Extremely satisfied (no, %)	29 (87.87%)
Satisfied (no, %)	4 (12.12%)
Acceptable (no, %)	0
Dissatisfied (no, %)	0

SD: standard deviation, EBSLN: external branch of superior laryngeal nerve, POD: postoperative day, and NIFTP: noninvasive follicular tumor with papillary-like nuclear features.

## Data Availability

The data used in this study are available at our registry (database) system at oncology center Mansoura University and upon request to the corresponding author Prof Dr Islam A. Elzahaby.
